# A case of radiotherapy for an advanced bronchial carcinoma patient with implanted cardiac rhythm machines as well as heart assist device

**DOI:** 10.1186/s13014-015-0378-8

**Published:** 2015-04-07

**Authors:** Sergiu Scobioala, Iris Ernst, Christos Moustakis, Uwe Haverkamp, Sven Martens, Hans Theodor Eich

**Affiliations:** Department of Radiotherapy and Radiooncology, University Hospital of Muenster, Muenster, 48149 Germany; Department of cardiac and thoracic surgery, University Hospital of Muenster, Muenster, 48149 Germany

**Keywords:** Pacemaker, Implantable cardioverter defibrillator, Cardiopulmonary support system, Helical tomotherapy, Stereotactic radiotherapy

## Abstract

We present a case of radiotherapy for a 66-year-old patient with squamous cell carcinoma on the left main bronchus undergoing implantation of pacemaker, implantable cardioverter defibrillator (ICD) as well as cardiopulmonary support (CPS) device. The radiation area was determined according to 4D List Mode positron emission tomography–computed tomography (PET-CT) data. Planning Target Volume (PTV) included a part of the active ICD. For the optimal tumor coverage and sparing of both the implantable cardiac devices and organs at risk, we combined the conformal radiotherapy with stereotactic body radiotherapy (SBRT) using helical tomotherapy. The prescription dose of 25.2Gy was applied by conventional radiotherapy. SBRT was performed hypofractionated with a prescription dose of 35Gy in 5 fractions. A dynamic electrocardiogram was performed during every radiation fraction. The implanted aggregates were checked three times a week. Despite partial localization of the active ICD in the radiation field, the tumor was treated without inappropriate shock delivery during radiation treatment and over twelve months afterwards. The reduced tumor size as well as tumor metabolic activity were observed by PET-CT three months after radiation treatment. The patient exhibited no signs of pneumonitis on the last radiological follow-up examination six months after radiotherapy. The reduced dyspnea and cough over the first four months after treatment were observed.

In conclusion, tumor shrinkage and temporary clinical improvement of the patient as well as no technical complications of implanted cardiac devices were achieved by the radiation treatment.

## Background

There are no large clinical studies for the determination of the exact radiation level that causes failure of the cardiac pacemaker and ICD. Most radiotherapy departments are using the radiation dose constraints for implanted devices based on the sparse number of the case reports and in vitro studies which occasionally demonstrate different results [1-3]. The American Association of Physicists in Medicine (AAPM) defined the guidelines for the irradiation exposure of the cardiac pacemakers in 1994 [4]. These guidelines are based on the first generation of Complementary-Metal-Oxide-Semiconductor (CMOS) pacemakers and do not take into account the newer radiation treatment technology. More recently, the Dutch guideline based on the newer data of in vitro experiments and several clinical studies was written [5]. These guidelines cover the recommendations for the irradiation of ICDs and consider the modern radiotherapy techniques such as intensity modulated radiation therapy (IMRT) or arc technique. Some investigators demonstrated a predisposition of batteries in modern cardiac pacemakers to quicker or even sudden unloading by an accumulated dose over 5Gy [3,6,7]*.* Radiation exposure of more than 2Gy is rarely found when the location of the heart pacemaker is outside the radiation field [8,9]. According to the data of Mouton et al. the failure of actually used CMOS pacemaker seems to appear at lower doses, also < 2Gy, but in a high dose rate [10]*.* Last et al. and Wilm et al. recommended to keep the cumulative exposure dose on heart pacemaker if possible <2Gy and by all means <10Gy, with the lowest possible dose rate [11,12]. ICDs are more sensitive to radiation than pacemakers because of scattered radiation effect on the random access memory (RAM) [13]. Manufacturers often provide the recommendations on radiation tolerance of their produced devices. However the recommended maximal dose tolerance differs considerably depending on the technical design of machines. For example, St.-Jude Medical GmbH® recommends the radiation dose limit for pacemaker by 20-30Gy and for ICD the dose tolerance was not stated [7,14]. The recommended dose limit for ICDs produced from Medtronic is variable from 1Gy to 5Gy depending on the model of ICD [7,15]. These recommendations do not take into consideration the recently used technical aspects and physical property of radiation treatment. In the literature we did not find any relevant information about radiation dose constraints or radiation resistance for the case of CPS device. We report here a possibility of the thoracic radiotherapy for central bronchial carcinoma patient with implanted cardiac pacemaker, ICD and assist device*. .* This case of radiation therapy is considered to be complicated because the active ICD is partially located in the radiation field.

## Patient and methods

**Figure 1:**
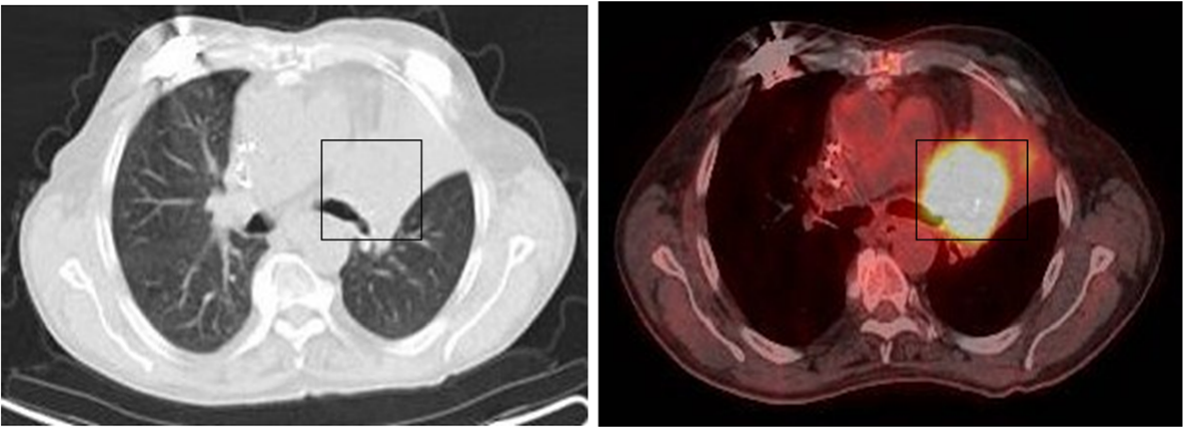
**Planning-PET-CT imaging by use of “fludeoxyglucose F 18” tracer.** Hypermetabolic activity of bronchial carcinoma on the left main bronchus with restriction of those.

A 66-year-old patient with squamous cell carcinoma, Grade II, on the left main bronchus, cT3 (5 cm) cN0 cM0 (Figure 1). A complete occlusion of the left upper lobe and partial involvement of the left lower lobe were detected by video-bronchoscopy*.* The general health condition was complicated with a mild dyspnea at rest and productive cough. The patient had initially undergone implantation of the CMOS-based cardiac pacemaker due to clinical relevant bradycardia developing on the basis of ischemic cardiomyopathy. The implanted cardiac pacemaker belongs to the “Accent” family pacemakers produced from St.-Jude Medical GmbH® (professional.sjm.com/products/crm). Six months later an implantation of the cardiac defibrillator was performed due to the first attack of the ventricular tachyarrhythmia. The ICD was activated shortly before radiation therapy due to a new attack of the tachyarrhythmia. The implanted ICD model – Atlas II VR SN – is the dual-chamber devices with automatic vector switching algorithm and algorithms for protection against inappropriate shock delivery (http://matesa.com.sv/manuales/atlasIIVR.pdf). A heart assist device, namely left ventricular assist devices (LVAD), was implanted two years later due to development of left heart failure NYHA III. The CPS device belongs to the long-term intracorporeal assisted system from company “Novacor” [16,17].

The radiation dose of the implanted devices was calculated before radiotherapy using a phantom measuring as well as during first three radiotherapy fractions by use of the thermoluminescent detector (TLD) for the control measurement.

**Figure 2:**
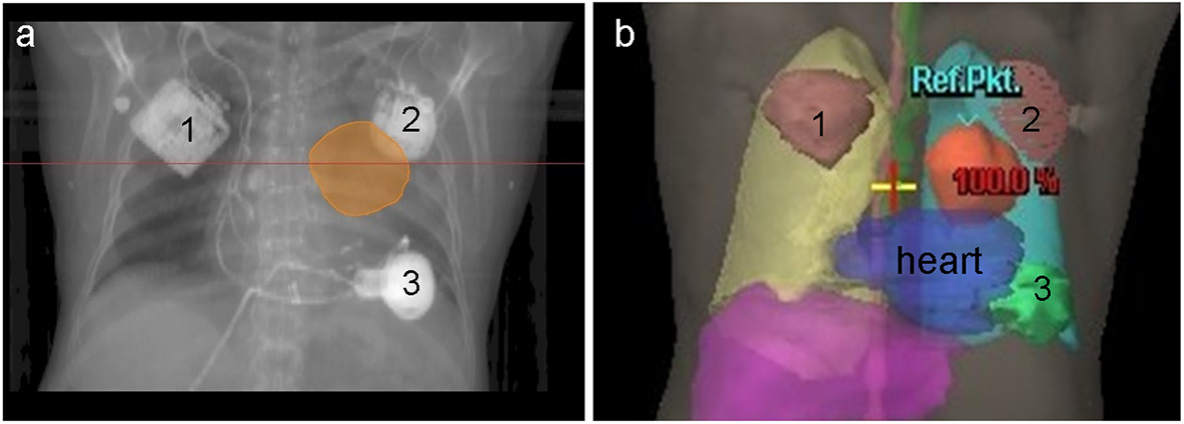
**PTV in relation to implanted cardiac devices: a) breathing- and heart beat-guided definition of PTV on the basis of 4D List Mode PET/CT; b) partial localization of the ICD in the radiation field.** 1- cardiac pacemaker, 2 – ICD, 3 – CPS device.

Target movement caused by breathing and heart beat was detected by 4D List Mode PET/CT (Figure 2). During this procedure the special integrated detector - Anzai Gating system on the Siemens camera - registered the motion amplitude of the indicated target during at least ten minutes. Each 4D PET scan was reconstructed into an ungated static image and analyzed by software [18,19].

**Figure 3:**
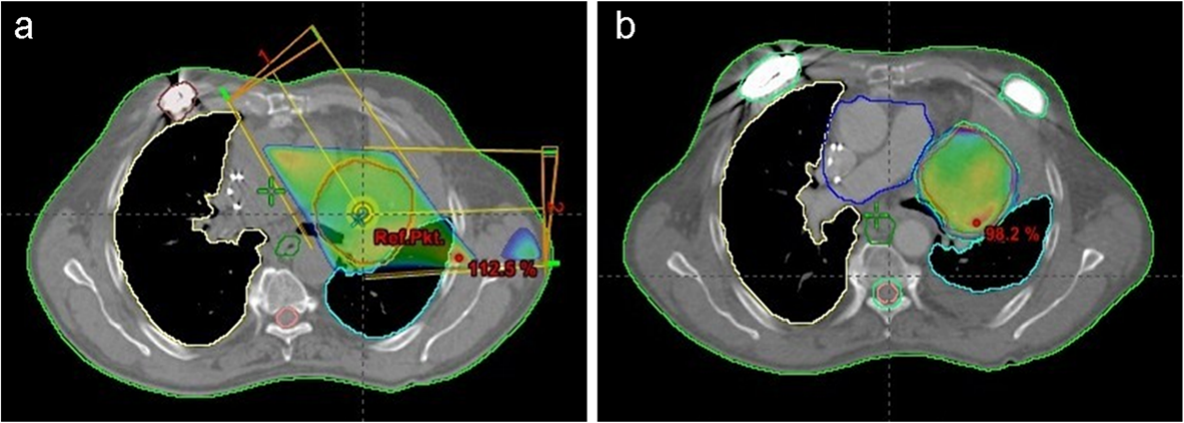
**Dose distribution in the radiation field:** **a) 2-field radiaton schedule prescribed to 95%- isodose line; (b) IMRT-based plan for SBRT prescribed to 65%-isodose line.**

2-field conformal radiotherapy prescribed to the 95% isodose line followed by stereotactic radiotherapy using helical tomotherapy and prescribed to the 65% isodose line were sequentially applied (Figure 3a, b)*.* The applied prescription dose of conformal radiotherapy is composed of 25.2Gy with 1.8Gy per fraction five times a week using 6/15 MV photon beams. Stereotactic radiotherapy was applied in a hypofractionated regime with the prescription dose of 35Gy and 7Gy per fraction three times a week by the use of 6MV photon beams.

The equipment that was maintained during radiation treatment included ‘crash cart’ with CPR devices and defibrillator with external pacemaker capacity. Electrocardiography (ECG) was performed during every treatment session. A technical control of cardiac pacemaker and defibrillators was performed three times a week during conventional radiotherapy and after every stereotactic fraction. After radiation treatment the implantable cardiac devices were checked weekly over the first month and then monthly in the cardiology department.

## Results

**Figure 4:**
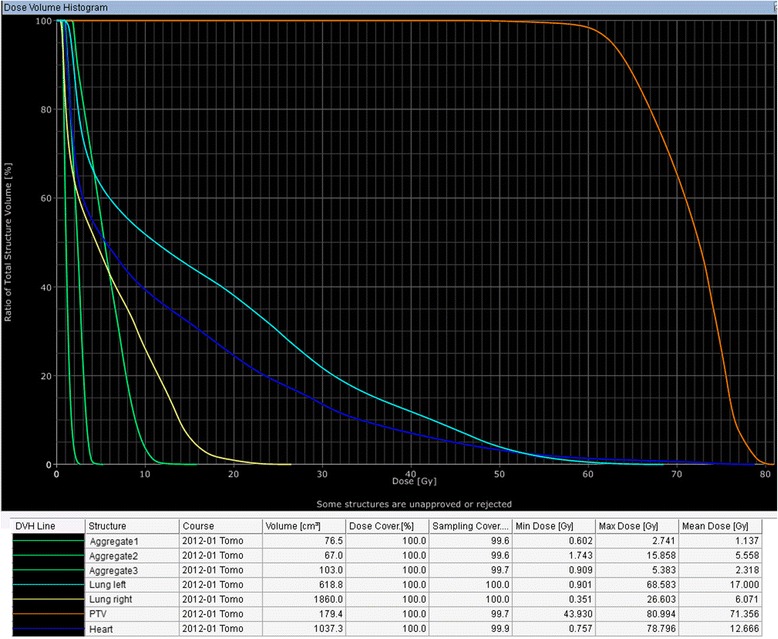
**Cumulative dose volume histogram.** Evaluation of the additive radiation dose in the target volume as well as in the implanted devices and organ at risk.

**Figure 5:**
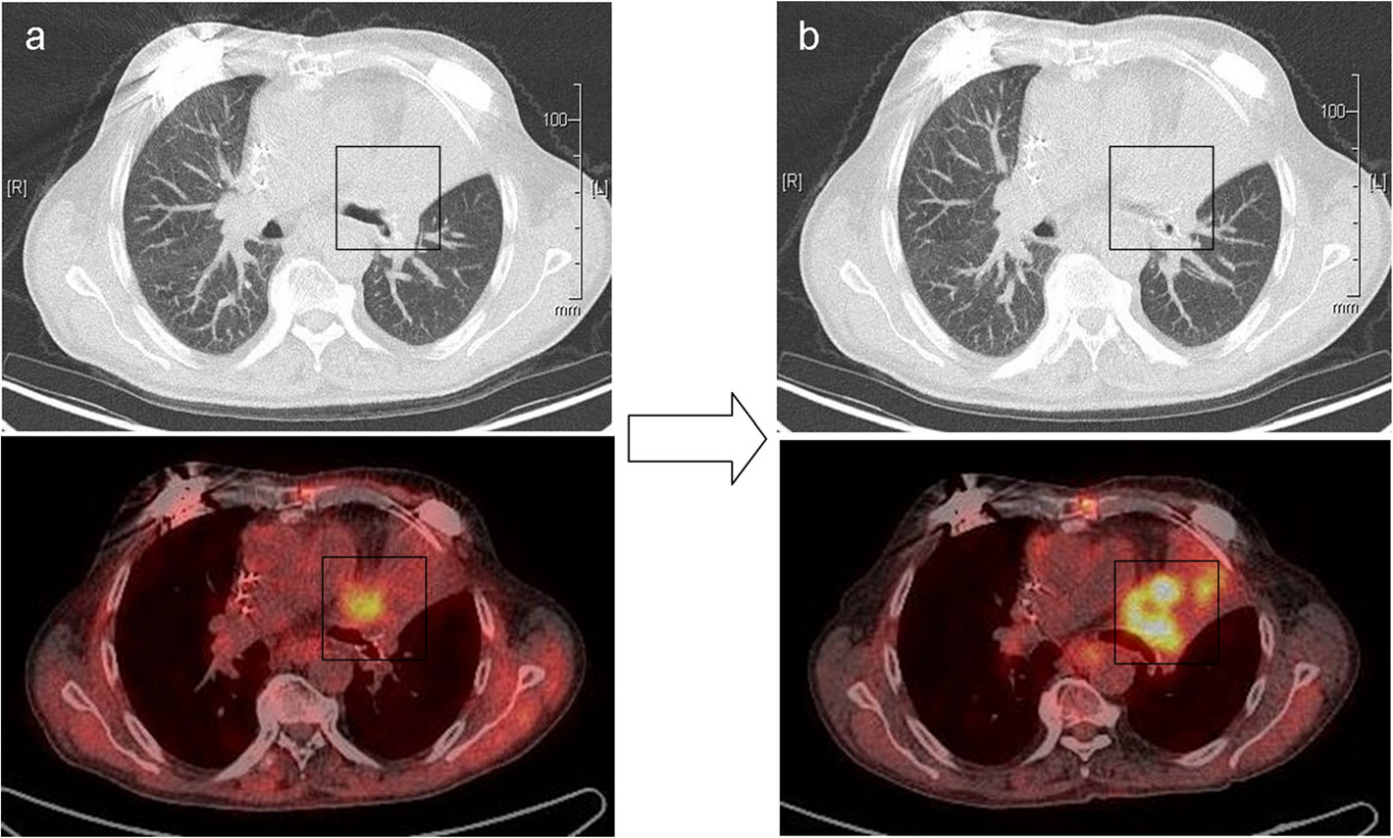
**Radiological follow-up staging after radiotherapy**: **a) significant reduction of tumor size and FDG-uptake in three months after radiotherapy; b) progressive disease in six months with the intrabronchial tumor propagation and increased metabolic activity.**

The maximal dose (Dmax) and median dose (Dmean) of the active ICD partially situated in the radiation field composed of 15.58Gy and 5.55Gy respectively. Dmax and Dmean of the CPS device and pacemaker located outside of the PTV composed of 5.38Gy/2.31Gy and 2.74Gy/1.13Gy respectively (Figure 4). Despite high radiation exposure we observed neither permanent nor temporary loss of function or inappropriate shock delivery by ICD. The implanted devices showed a non-affected function during radiation treatment and over the course of twelve months afterwards. The radiation treatment had generally no negative impact on the patient's cardiac rhythm and blood circulation parameters. The treatment response correlated with reduction of tumor size and tumor metabolic activity as demonstrated three months after radiotherapy by PET-CT (Figure 5a). Therefore, the reduction of the maximal “standardized uptake value” (SUV) in PET scans from pretreatment level of 14,8 to 6,1 was observed three months after radiation therapy. Six months post radiation treatment a progressive disease with new intrabronchial growth and enhanced metabolic activity of primary tumor by maximal SUV 12,9 was verified (Figure 5b).

The mean radiation dose for the ipsilateral lung composed of 17.00Gy, and V_20_ demonstrated nearly 40% in the dose volume histogram (40% of lung volume with radiation dose of 20Gy) (Figure 4). This is high radiation lung toxicity with the probability for radiation induced pneumonitis from 30 to 40% [20,21]. On the other hand, a forced expiratory pressure in 1 s (FEV_1_) of 75,9% (2,23 L) from reference parameter in the pretherapeutical spirometry pointed to sufficient pulmonary reserve by the patient. According to our data the aggravated risk of pneumonitis may develop by pretherapeutical FEV_1_ under 60% from reference parameter. Despite relative high risk for the radiation induced pneumonitis, no clinical and radiological signs of those were detected in the course of six months after radiotherapy (the last radiological follow-up examination) (Figure 5b). Moreover, the patient exhibited a reduced dyspnea and cough over the first four months after treatment. Sixteen months after radiation treatment the patient died due to lung insufficiency induced by tumor progression with the developing of massive ipsi- and contralateral pulmonal metastases.

## Discussion

An impact of modern radiotherapy techniques or electromagnetic fields of linear accelerators, as well as dose rate effects on the frequency and cause of device failures are actually relevant to consider. So, the use of IMRT technique might lead to less radiation dose to pacemakers because of reduced use of high energy photon beams [5]*.* At the same time, it is a prolonged radiotherapy treatment with more additional fields for a better dose distribution, what might produce the malfunction [3,7,22]. Hashii et al. and Gelblum et al. demonstrated in vitro the higher neutrons production by use of beam energies above 10 MV, what seem to generate the function defects of pacemakers [23,24]. Elders et al. and Zaremba et al. observed the same correlation between the ICDs malfunction and photon beam energies ≥10 MeV in vivo studies. The authors hypothesized that this correlation may be based on the interaction of neutrons produced in the head of the linear accelerator at higher beam energies with boron situated in the internal circuitry of ICD [25,26]. In 2002 Mouton et al. published the largest study involved *in vitro* examination of dose rate effects on pacemaker's functionality [10]. No defects at the dose rate of 0.2 Gy/min. were observed on the pacemakers studied. One could consider this as the maximum acceptable dose rate.

The combination of SBRT and conventional radiotherapy was applied for more effective local tumor control via stereotactic radiotherapy. On the other hand, the conventional radiation treatment is more suitable for the sparing of the organ at risk especially for organ with the slower cell turnover and lower α/β value, such as heart and lung [27]. Of course, it is not fully correct to directly summarize the doses from two different types of radiation with different biological effectiveness. To our knowledge, there are no calculation factors to relativize the hypofractional radiotherapy to conventional fractionated radiation treatment. As the next step, we plan to introduce the special weighting factors to evaluate more precisely the cumulative dose and respectively the summed biological effect by the combination of conventional and hypofractional radiotherapy.

On the basis of some new clinical studies, the Dutch guideline categorizes the radiotherapy patients into low, medium and high risk groups [5]. The principal criterions for this clinical classification are cumulative radiation dose to pacemakers and pacing dependency of patients. Patients with the radiation dose of less than 2 Gy are categorized as low risk unless they are pace dependent. The medium risk category consists of patients with doses between 2 and 10Gy, and the dose above 10Gy categorizes all patients of high risk group. This risk dependent classification gives some practical guidelines for the management of patients having the cardiac rhythm machines. So, a cardiac monitoring (ECG) during every treatment session and device checks within 24 hours after every fraction are needed to be done when the cumulative dose is between 2Gy and 10Gy. For doses above 10Gy a relocation of the pacemakers should be discussed. It is recommended to monitor the pacemaker's functionality for at least the first six months after radiation treatment [5,7].

It is necessary to calculate the dose received by the pacemaker or ICD before radiation treatment. The use of more modern planning system allows evaluating the received dose in the radiation treatment plan by dose-volume histograms. According to AAPM guideline a thermoluminescent dosimetry (TLDs) must be performed on day one to check the dose received by the devices. Before radiotherapy, we performed a phantom measuring in order to calculate the potential irradiation dose for all implanted devices. Additionally, a TLD measuring was performed during the first three radiation sessions in order to control the received dose. Data from these measures demonstrated radiation tolerance of the ICD with a maximal dose of 15.85 Gy. The generally recommended safety measures in terms of ICD as reprogramming of deactivating or applying a heavy magnet for the prevention of an inappropriate shock delivery was not used during the radiation treatment. Actually there are controversial opinions concerning the effectiveness of these procedures and these subjects should be considered in further examinations [5,28,29]. In our case, the patient had an acute indication for radiation treatment because of the rapid tumor progress. On the other side, the new attack of the ventricular tachyarrhythmia shortly before radiotherapy has occurred. In our opinion, it would be more perilous to inhibit the antitachycardia therapy in this situation. There are a little clinical data about the impact of the hypofractionated schedules on the functionality of cardiac rhythm devices. The higher fraction dose might have a higher probability of the radiation induced malfunction. Although, using IMRT based treatment a radiation exposure on devices can be reduced by low energy photon beams. So, an SBRT with 7Gy fraction dose in our case was applied using tomotherapie with 6MV beam energies without any technical dysfunction of pacemaker or ICD, or any clinical consequences for the patient. In addition, the helical tomotherapy schedule demonstrated an optimal ratio between the tumor coverage and sparing of the cardiac implantable machines. Both AAPM and Dutch guidelines recommended managing the dose distribution without direct involvement of the electronic devices or even with few centimeters distance of those from the radiation field. In case of potentially high radiation dose, the relocation of devices should be considered. The relocation for our patient would be technically complicated due to tumor expansion on implanted ICD (Figure 2). Moreover, a clinical aggravation of the patient with rapidly developing dyspnea at rest required a sooner beginning of the palliative treatment. For this reason a high radiation exposure of 15.85Gy (Dmax) was inevitable due to partial localization of ICD in radiation area. ECG during every radiotherapy session as well as technical control of devices three times a week did not reveal any dysfunction of implanted machines. Also, no device failures such as inappropriate shock delivery were observed at all over the course of one year after radiotherapy. To our knowledge, there is no available data about radiation tolerance of the CPS device as well as influence of the electric and magnetic fields of linear accelerators to the functionality of assist machine. Interestingly, the high radiation exposure of the CPS machine with the maximal radiation dose of 5,38Gy (Dmax) had no negative impact on cardiac and blood circulation parameters in our case.

In order to achieve the optimal covering of tumor area by minimal radiation exposure of the implantable electronic rhythm devices and CPS machine, a stereotactic radiotherapy schedule was evaluated. For this purpose, a helical tomotherapy (Accuray Inc®, USA) and Intensity Modulated Arc Therapy (IMAT) for TrueBeam LINAC (Varian Medical Systems®, USA) were scheduled. Tomotherapy demonstrated better tumor coverage as well as sparing of implanted machines despite a little increased radiation dose of ipsi- and contralateral lung*.* The reduced lung toxicity was minimized by 2-field conformal radiotherapy. Thus, the combination of stereotaxic hypofractional radiotherapy with conformal external beam radiation was able to deliver the optimal dose distribution with sparing all risk structures.

## Conclusions

The presented case demonstrates both a temporal clinical improvement of the palliative patient as well as high radiation tolerance of ICD partially located in radiation area. Despite extremely high radiation dose to ICD, no dysfunction such as inappropriate shock delivery was observed at all. The demonstrated radiation treatment is based on individual concept. This therapy was only possible through multidisciplinary team of expert cardiologists, medical physicists and radiation oncologists.

## Consent

A written informed consent was obtained from the patient’s wife for publication of this case report and any accompanying images. A copy of the written consent is available for review by the Editor-in-Chief of this journal.

## Abbreviations

AAPM: American Association of Physicists in Medicine
CMOS: Complementary-Metal-Oxide-Semiconductor
CPS: Cardiopulmonary support
SBRT: Stereotactic body radiotherapy
ECG: Electrocardiography
FEV_1_: Forced expiratory pressure in 1 s
ICD: Implantable cardioverter defibrillator
IMAT: Intensity Modulated Arc Therapy
IMRT: Intensity modulated radiation therapy
LVAD: Left ventricular assist devices
PTV: Planning Target Volume
RAM: Random access memory
SUV: Standardized uptake value
TLD: Thermoluminescent dosimetrys

## References

[1] Dorenkamp M, Stromberger C, Von Heymann C, Haverkamp W, Wust P, Roser M (2013). Ra-diation therapy in patients with cardiac pacemakers or implantable cardioverter defibrillators. Interdisciplinary safety recommendations. Strahlenther Onkol.

[2] Munshi A, Agarwal JP, Pandey KC (2013). Cancer patients with cardiac pacemakers needing radiation treatment: a systematic review. J Cancer Res Ther.

[3] Lambert P, Da Costa A, Marcy PY, Kreps S, Angellier G, Marcié S (2011). Pacemaker, implanted cardiac defibrillator and irradiation: Management proposal in 2010 depending on the type of cardiac stimulator and prognosis and location of cancer. Cancer Radiother.

[4] Marbach JR, Sontag MR, Van Dyk J, Wolbarst AB (1994). Management of radiation oncology patients with implanted cardiac pacemakers: report of AAPM Task Group No. 34. American Association of Physicists in Medicine. Med Phys.

[5] Hurkmans CW, Knegjens JL, Oei BS, Maas AJJ, Uiterwaal GJ, Van der Borden AJ (2012). Lieselot van Erven L: Management of radiation oncology patients with a pacemaker or ICD: A new comprehensive practical guideline in The Netherlands. Radiat Oncol.

[6] Röthig H, Herrmann T, Kopcsek H (1995). Experience in dealing with artificial pacemaker patients during therapy with ionizing radiation. Strahlenther Onkol.

[7] Hudson F, Coulshed D, D'Souza E, Baker C (2010). Effect of radiation therapy on the latest generation of pacemakers and implantable cardioverter defibrillators: A systematic review. J Med Imaging Radiat Oncol.

[8] Sundar S, Symonds RP, Deehan C (2005). Radiotherapy to patients with artificial cardiac pacemakers. Cancer Treat Rev.

[9] Solan AN, Solan MJ, Bednarz G, Goodkin MB (2004). Treatment of patients with cardiac pacemakers and implantable cardioverter-defibrillators during radiotherapy. Int J Radiat Oncol, Biol, Phys.

[10] Mouton J, Haug R, Bridier A, Dodinot B, Eschwege F (2002). Influence of high-energy photon beam irradiation on pacemaker operation. Phys Med Biol.

[11] Last A (1998). Radiotherapy in patients with cardiac pacemakers. Br J Radiol.

[12] Wilm M, Kronholz HL, Schütz J, Koch T (1994). The modification of programmable pacemakers by therapeutic irradiation. Strahlenther Onkol.

[13] Guidant Corp (2004). Impact of Therapeutic Radiation and Guidant ICD/CRT-p/Pacing Systems Review. European Technical Services, St Paul, MN.

[14] St. Jude Medical. Radiation. 1-3, 9/02 and Electromagnetic Interference. 1-6, no date, Cardiac Rhythm Management Division, Sylmar, CA.

[15] Medtronic Incorporated (2008). Therapeutic Radiation.

[16] El-Banayosy A, Deng M, Loisance DY, Vetter H, Gronda E, Loebe M (1999). The European experience of Novacor left ventricular assist (LVAS) therapy as a bridge to transplant: a retrospective multi-centre study. Eur J Cardiothorac Surg.

[17] Di Bella I, Pagani F, Banfi C, Ardemagni E, Capo A, Klersy C (2000). Results with the Novacor assist system and evaluation of long-term assistance. Europ J of Cardio-thoracic Surg.

[18] Callahan J, Binns D, Dunn L, Kron T (2011). Motion effects on SUV and lesion volume in 3D and 4D PET scanning. Australas Phys Eng Sci Med.

[19] Van Elmpt W, Hamill J, Jones J, De Ruysscher D, Lambin P, Ollers M (2011). Optimal gating compared to 3D and 4D PET reconstruction for characterization of lung tumours. Eur J Nucl Med Mol Imaging.

[20] Kwa SL, Lebesque JV, Theuws JC, Marks LB, Munley MT, Bentel G (1998). Radiation pneumonitis as a function of mean lung dose: an analysis of pooled data of 540 patients. Int J Radiat Oncol, Biol, Phys.

[21] Byhardt RW, Martin L, Pajak TF, Shin KH, Emami B, Cox JD (1993). The influence of field size and other treatment factors on pulmonary toxicity following hyperfractionated irradiation for inoperable non-small cell lung cancer (NSCLC)–analysis of a Radiation Therapy Oncology Group (RTOG) protocol. Int J Radiat Oncol, Biol, Phys.

[22] Ferrara T, Baiotto B, Malinverni G, Caria N, Garibaldi E, Barboni G (2010). Irradiation of pacemakers and cardio-defibrillators in patients submitted to radiotherapy: a clinical experience. Tumori.

[23] Hashii H, Hashimoto T, Okawa A, Shida K, Isobe T, Hanmura M (2013). Comparison of the effects of high-energy photon beam irradiation (10 and 18 MV) on 2 types of implantable cardioverter-defibrillators. Int J Radiat Oncol, Biol, Phys.

[24] Gelblum DY, Amols H (2009). Implanted cardiac defibrillator care in radiation oncology patient population. Int J Radiat Oncol, Biol, Phys.

[25] Elders J, Kunze-Busch M, Jan Smeenk R, Smeets JL (2013). High incidence of implantable cardioverter defibrillator malfunctions during radiation therapy: neutrons as a probable cause of soft errors. Europace.

[26] Zaremba T, Jakobsen AR, Thøgersen AM, Oddershede L, Riahi S (2014). The effect of radiotherapy beam energy on modern cardiac devices: an in vitro study. Europace.

[27] Wannenmacher M, Debus J, Strahlentherapie WF, Weber K-J, Wenz F (2006). Chapter 2: K.-J. Strahlenbiologische Grundlagen.

[28] Sears SF, Hauf JD, Kirian K, Hazelton G, Conti JB (2011). Posttraumatic stress and the implantable cardioverter-defibrillator patient: what the electrophysiologist needs to know. Circ Arrhythm Electrophysiol.

[29] Habibovic M, Van den Broek KC, Alings M, Van der Voort PH, Denollet J (2012). Posttraumatic stress 18 months following cardioverter defibrillator implantation: shocks, anxiety, and personality. Health Psychol.

